# Primary cilium remodeling mediates a cell signaling switch in differentiating neurons

**DOI:** 10.1126/sciadv.abb0601

**Published:** 2020-05-20

**Authors:** Gabriela Toro-Tapia, Raman M. Das

**Affiliations:** Division of Molecular and Cellular Function, Faculty of Biology Medicine and Health, University of Manchester, Michael Smith Building, Oxford Road, Manchester M13 9PT, UK.

## Abstract

Cellular differentiation leads to the formation of specialized cell types and complex morphological variations. Often, differentiating cells transition between states by switching how they respond to the signaling environment. However, the mechanisms regulating these transitions are poorly understood. Differentiating neurons delaminate from the neuroepithelium through the regulated process of apical abscission, which mediates an acute loss of polarity and primary cilium disassembly. Using high-resolution live-cell imaging in chick neural tube, we show that these cells retain an Arl13b^+^ particle, which elongates and initiates intraflagellar trafficking as it transits toward the cell body, indicating primary cilium remodeling. Notably, disrupting cilia during and after remodeling inhibits axon extension and leads to axon collapse, respectively. Furthermore, cilium remodeling corresponds to a switch from a canonical to noncanonical cellular response to Shh. This work transforms our understanding of how cells can rapidly reinterpret signals to produce qualitatively different responses within the same tissue context.

## Teaser

Newborn neurons assemble a new primary cilium, leading to a change in the mode of Shh signaling that now directs axon extension.

## INTRODUCTION

The early embryonic neuroepithelium consists of elongated neural progenitor cells that form membrane contacts with neighboring cells at both their apical and basal poles (*1*). These cells have a microtubule-based primary cilium, which extends from the apical membrane, forming a specialized compartment that is required for transduction of canonical, Gli transcription factor–dependent Shh signaling (*2*). In this context, canonical Shh signaling maintains neuroepithelial cells as cycling progenitors. As these cells begin neuronal differentiation, they localize their cell bodies toward their basal poles and delaminate from the neuroepithelium. This process involves loss of apical polarity and primary cilium disassembly through the regulated process of apical abscission, which is followed by retraction of the apical process (*3*). This step correlates with cell cycle exit through cessation of canonical Shh signaling. Following this, newborn neurons switch to noncanonical, transcription factor–independent Shh signaling, which now mediates axon navigation (*4*, *5*), a process that is critical for the formation of functional neural circuitry. Although this switch in cell signal interpretation is well known, the cellular mechanisms directing it remain unknown.

Primary cilia play important roles in integrating and transducing extracellular signals (*6*), and many neuronal subtypes have primary cilia (*7*). Here, using live tissue imaging and high-resolution fixed tissue imaging, we reveal a previously unidentified mechanism that mediates the switch from canonical to noncanonical Shh signaling. We show that primary cilium disassembly through apical abscission leads to acute cessation of canonical Shh signaling. This is followed by rapid remodeling of the primary cilium to build a new signaling center with a molecular configuration that is distinct from the original primary cilium. This new configuration facilitates the switch to noncanonical Shh signaling, which now directs the process of axon extension. Cilium remodeling therefore provides differentiating neurons the opportunity to configure a new signaling center to facilitate a qualitatively different response to the common signaling environment.

## RESULTS

### Delaminating neurons undergo rapid primary cilium reassembly

**Fig. 1:**
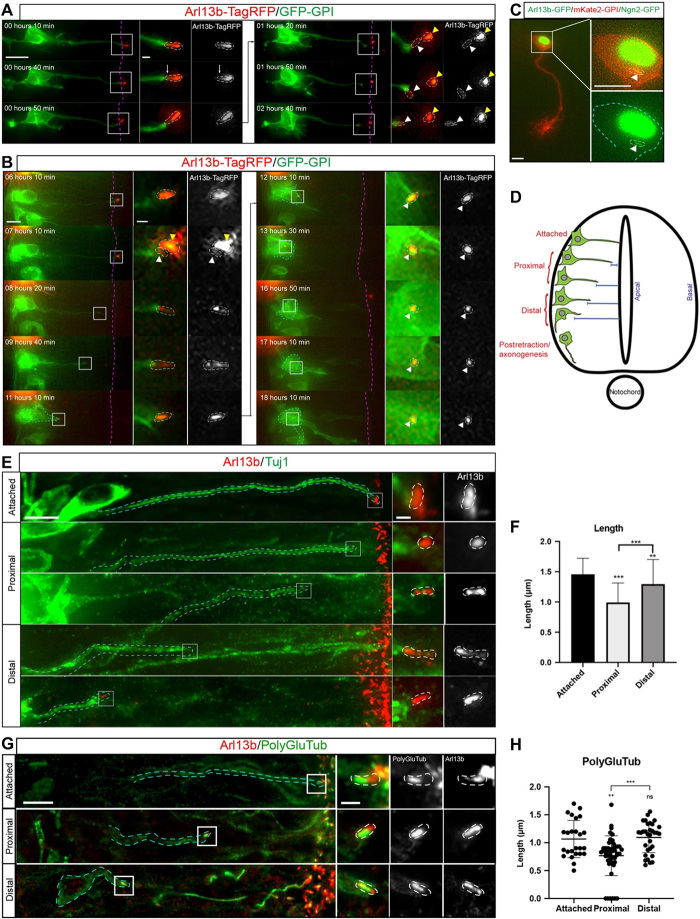
The primary cilium is dynamically disassembled and reassembled during neuronal differentiation. (**A**) Time-lapse sequence showing Arl13b^+^ particle retention following apical abscission (movie S1). White arrows indicate apical abscission, white arrowheads indicate the retained Arl13b^+^ particle, and yellow arrowheads indicate the abscised primary cilium. Dashed magenta lines indicate ventricular surface. Scale bar, 10 μm. Boxed regions are enlarged in right-hand panels. Primary cilium is highlighted by white dashed lines. Scale bar, 0.7 μm. (**B**) Time-lapse sequence of a cell undergoing primary cilium reassembly during apical process retraction (movie S2). Cyan dashed lines demarcate cell body of differentiating neuron, and white arrowheads indicate the reassembling primary cilium. Boxed regions are enlarged in right-hand panels. Scale bars, 5 and 0.7 μm (enlarged region). (**C**) Cell undergoing axonogenesis transfected with Arl13b-GFP to label the ciliary membrane (green), mKate2-GPI to label cell membrane (red), and Neurog2 (Ngn2-GFP, green) (movie S3). Scale bars, 5 μm. (**D**) Representation of differentiating neurons at different stages of apical process retraction and early stages of axonogenesis. (**E**) Immunostaining to detect Tuj1^+^ cells (green) and endogenous Arl13b (red) at different stages of apical process retraction. Scale bars, 5 and 0.7 μm (enlarged region). (**F**) Quantification of absolute length of primary cilia (μm) from proximal-distal stages of apical process retraction in fixed tissue [mean ± SD, **P < 0.01 and ***P < 0.001, ordinary one-way analysis of variance (ANOVA) and Tukey’s post hoc test used for statistical analyses]. (**G**) Immunostaining to detect endogenous Arl13b and polyglutamylated tubulin (PolyGluTub) at different stages of apical process retraction. (**H**) Quantification of absolute length of the PolyGlu-labeled axoneme from proximal-distal stages of apical abscission in fixed tissue (mean ± SD, **P < 0.01 and ***P < 0.001, ordinary one-way ANOVA and Tukey’s post hoc test used for statistical analyses). ns, not significant.

To investigate cell behavior following primary cilium disassembly by apical abscission (*3*), we labeled membranes of individual neuroepithelial cells by mosaic transfection of green fluorescent protein (GFP)–glycosylphosphatidylinositol (GPI) and the primary cilium membrane marker Arl13b-TagRFP (*8*). We then monitored neuronal differentiation in ex vivo embryo slice cultures using wide-field time-lapse microscopy (*9*). Unexpectedly, this revealed that while most of the ciliary membrane is shed during apical abscission, a small portion of this was retained by the withdrawing apical process (Fig. 1A, *n* = 46 of 51 cells, 15 embryos, movie S1). This retained Arl13b^+^ particle remained at the tip of the apical process and progressively elongated as apical process withdrawal proceeded and was then closely associated with the neuronal cell body as axonogenesis initiated, indicating that the Arl13b^+^ particle acts as a site for primary cilium reassembly (Fig. 1B, *n* = 39 of 46 cells, 15 embryos, movie S2; Fig. 1C, *n* = 25 cells, 10 embryos, movie S3). Cells in early stages of apical process retraction were classified as proximal to the apical surface, while those in later stages of retraction were classified as being distal to the apical surface (Fig. 1D). Quantification of the relative length of the reassembling primary cilium revealed that the Arl13b^+^ particle was approximately 50% of the length of the original primary cilium, and this increased to 75% toward completion of apical process retraction (fig. S1A; *n* = 6, 6 embryos). To confirm these observations in cells that are not misexpressing Arl13b, which can lead to inconsistencies in cilium length (*10*), and in tissue not subjected to our live imaging regime, we fixed and labeled completely unmanipulated embryos with the early neuronal marker Tuj1 and Arl13b (Fig. 1E). Similar to our previous results, proximal cells that had recently undergone apical abscission had a short Arl13b^+^ particle (Fig. 1F; 0.99 ± 0.32 μm, *n* = 190 cells, 14 embryos), and this structure progressively elongated in distal cells (Fig. 1F; 1.29 ± 0.40 μm, *n* = 82 cells, 14 embryos), localizing at the tip of the apical process. To further characterize primary cilium reassembly, we labeled unmanipulated embryos with Tuj1 and polyglutamylated tubulin to visualize the ciliary axoneme (Fig. 1G). This revealed that proximal cells, which had recently undergone apical abscission, also had a short axoneme localized within the retained Arl13b^+^ particle (Fig. 1H; 0.76 ± 0.36 μm, *n* = 48 cells, 8 embryos), which progressively elongated in distal cells (Fig. 1H; 1.09 ± 0.27 μm, *n* = 32 cells, 8 embryos). Furthermore, the ratio between the length of the axoneme and Arl13b remained constant throughout apical process retraction, indicating that the axoneme is reassembled proportionally to the ciliary membrane (fig. S1B). Together, these results show that primary cilia of newborn neurons undergo disassembly during apical abscission, followed by rapid reassembly during apical process retraction. Furthermore, the reassembled primary cilium is maintained in cells undergoing axonogenesis.

### Intraflagellar transport is progressively reinitiated during apical process retraction

**Fig. 2:**
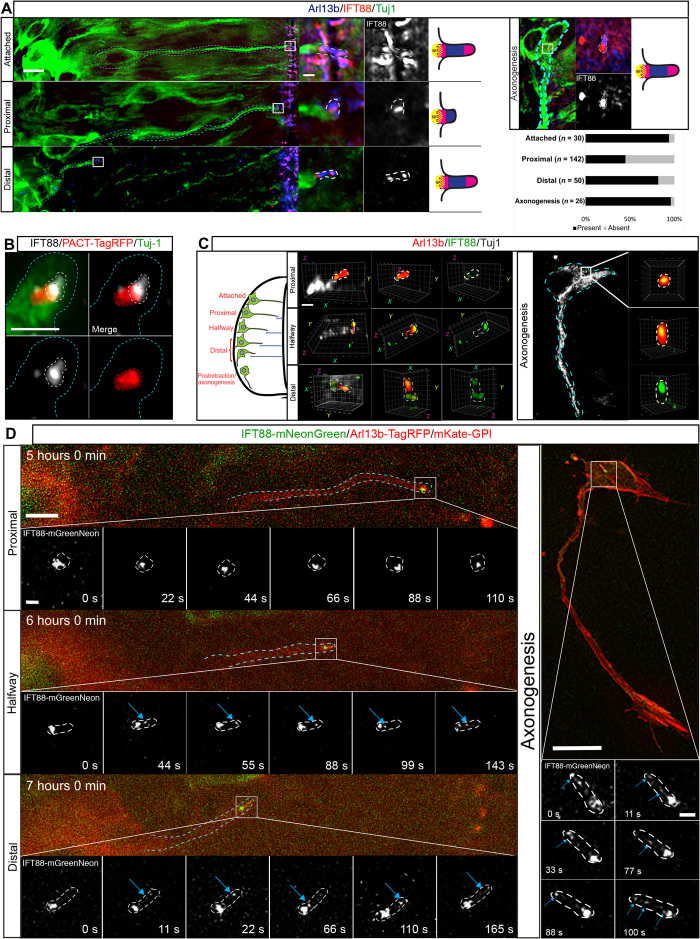
Intraflagellar trafficking is inactive in the Arl13^+^ particle and reactivated during apical process retraction. (**A**) Immunostaining to detect endogenous IFT88 (red), Arl13b (blue), and Tuj-1 (green). Cyan dashed lines demarcate differentiating cells, and boxed regions are enlarged in right-hand panels followed by a cartoon of representative IFT88 localization (BB, basal body). Primary cilium is highlighted by white dashed lines. Top right panels show primary cilium in cell body of Tuj1^+^ cell extending axon in the basal region of the spinal cord. Bottom right panel shows quantification of percentage of cells with detectable IFT88 in ciliary tip (present) at different stages of apical process retraction and axonogenesis. Scale bars, 5 and 0.7 μm (enlarged region). (**B**) Immunofluorescence and Airyscan enhanced-resolution microscopy of misexpressed PACT-TagRFP (red) and endogenous IFT88 (white). (**C**) Airyscan 3D reconstruction of primary cilia (red) at different stages of apical process retraction (stages are summarized in the diagram on the left; movies S4 to S7). Cyan dashed lines demarcate the tip of apical process, red dashed lines demarcate the centrosome, and white dashed lines demarcate the remodeled cilium. Scale bar, 1 μm. (**D**) Time-lapse sequence of differentiating neuron misexpressing IFT88-mNeonGreen undergoing apical process retraction (movie S8) and axonogenesis (movie S9). Cyan dashed lines outline differentiating cell. Enlarged regions indicate representative still images from time-lapse sequences. Blue arrows indicate IFT88 in reassembled primary cilium. Scale bars, 5 μm (process retraction), 10 μm (axonogenesis), and 0.7 μm (enlarged regions).

Intraflagellar transport (IFT) is required for trafficking of proteins involved in assembly and maintenance of the primary cilium and to transport proteins that transduce cilium-mediated signaling (*11*, *12*). To further investigate primary cilium functionality during reassembly, we labeled unmanipulated embryos for Tuj1 and a member of the anterograde IFT complex, IFT88 (*11*, *13*). Tuj1^+^ cells that were attached to the apical surface presented high levels of IFT88 at the tips of their primary cilia (Fig. 2A; *n* = 31 of 33 cells, 12 embryos), indicating active IFT in these cells. In contrast, IFT88 did not localize to the tip of the retained Arl13b^+^ particle but was frequently detected only in the basal region in cells proximal to the apical surface (Fig. 2A; *n* = 78 of 142 cells, 12 embryos). This IFT88 partially colocalized with the basal body labeled with PACT-TagRFP (Fig. 2B; *n* = 41 of 55 cells, 6 embryos), suggesting inactive IFT at these stages, with a reservoir of IFT88 at the base of the cilium to facilitate rapid reactivation of IFT (*14*, *15*), which is essential for ciliogenesis. Consistent with this, IFT88 notably reappeared in tips of primary cilia of cells distal to the apical surface (Fig. 2A; *n* = 41 of 50 cells, 12 embryos), suggesting reinitiation of IFT in reassembled primary cilia. This pattern of IFT88 localization was maintained in cells undergoing axonogenesis (Fig. 2A; *n* = 25 of 26 cells, 9 embryos), suggesting maintenance of IFT in this cell population. This progressive restoration of ciliary IFT88 accumulation as neuronal differentiation progresses was confirmed by three-dimensional (3D) Airyscan microscopy (Fig. 2C and movies S4 to S7). This revealed in detail IFT88 at the basal region of the retained Arl13b^+^ particle in cells proximal to the apical surface and an evident accumulation in the tips of reassembled primary cilia of cells that had retracted halfway or were distal to the apical surface and in cells undergoing axonogenesis. These results strongly suggest a cessation of IFT in the retained Arl13b^+^ particle following apical abscission, which is progressively restored as the cilium elongates, implying that this structure is remodeled in newborn neurons.

To confirm progressive reinitiation of IFT during primary cilium remodeling, we then monitored the temporal dynamics of IFT88 localization during apical process retraction. Neuroepithelial cells were cotransfected with IFT88-mNeonGreen, Arl13b-TagRFP, and mKate2-GPI, and cells that had recently undergone apical abscission were rapidly imaged for 3 min every hour, allowing us to capture the key stages of apical process retraction (Fig. 2D; *n* = 6 cells, 4 embryos; movie S8). This revealed that in proximal cells, IFT88 was relatively static and remained localized to the base of the Arl13b^+^ particle but did not colocalize with Arl13b, indicating inactive IFT. Halfway through retraction, as the retained particle elongated, IFT88 trafficking to the tip of the primary cilium was reestablished, indicating that primary cilium–mediated signal transduction is also reactivated at this point, as IFT complexes are required for proper dynamics of diverse signaling components within the primary cilium (*16*, *17*). IFT88 trafficking was then maintained in distal cells and in cells undergoing axonogenesis (Fig. 2D, right-hand panels; *n* = 6 cells, 5 embryos; movie S9). Consistent with these observations, quantification of IFT88 present within the primary cilium revealed a progressive increase in the area occupied by IFT88 in the primary cilium as well as a progressive increase in the number of IFT88 particles present in the primary cilium as cells retracted their apical processes (fig. S2). These results confirm that IFT is progressively restored as newborn neurons delaminate from the neuroepithelium and indicate that the molecular machinery required for signal transduction is reestablished during the later stages of apical process retraction.

### The remodeled primary cilium directs initiation and maintenance of axonogenesis

**Fig. 3:**
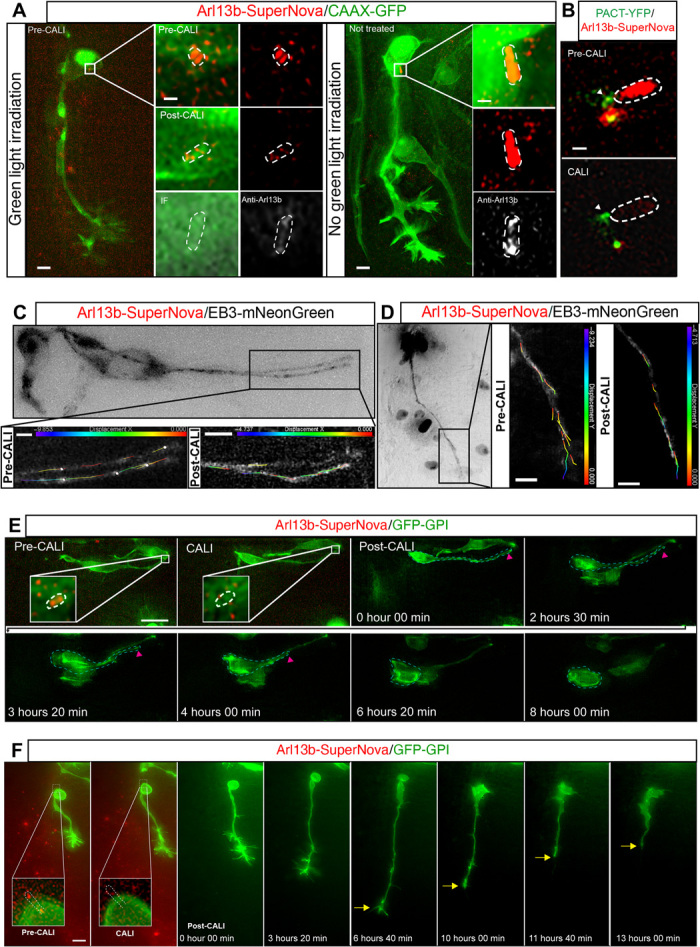
The remodeled primary cilium is essential for establishment and subsequent maintenance of the nascent axon. (**A**) Immunostaining to detect Arl13b (white) in cells subjected and not subjected to green light irradiation. Boxed regions are enlarged in right-hand panels. Scale bars, 5 and 0.7 μm (enlarged region). IF, immunofluorescence. (**B**) PACT–YFP (yellow fluorescent protein) and Arl13b-SuperNova were misexpressed to label the centrosome in CALI experiments (movie S10). GFP fluorescence was compared before and after green light stimulation. Arrowhead indicates the centrosome. Scale bar, 0.7 μm. (**C**) Cell attached to the apical surface misexpressing Arl13b-SuperNova and EB3-mNeonGreen to label microtubule plus-ends (movie S11). Comets were tracked before and after CALI. Boxed regions are enlarged in bottom panels (before and after CALI) showing displacement of individual EB3-GFP comets. Scale bar, 5 μm. (**D**) Cell undergoing axonogenesis misexpressing Arl13b-SuperNova and EB3-mNeonGreen (movie S12). Comets were tracked before and after CALI. Boxed regions are enlarged in bottom panels (before and after CALI) showing displacement of individual EB3-GFP comets. (**E**) Time-lapse sequence of a cell undergoing apical process retraction following CALI-mediated disruption of the retained Arl13b^+^ particle (movie S13). Cyan dashed lines demarcate the differentiating cell, and the inset shows a zoomed-in view of the retained Arl13b^+^ particle highlighted by white dashed lines. Magenta arrowhead indicates the tip of the retracting process. Scale bar, 10 μm. (**F**) Time-lapse sequence of CALI-mediated disruption of primary cilium in cell undergoing axonogenesis and subsequent cell behavior. The inset shows a zoomed-in view of the primary cilium highlighted by white dashed lines. Yellow arrows indicate axon collapse (movie S14). Scale bar, 10 μm.

To investigate whether reestablishment of primary cilium–mediated signal transduction is required for neuron repolarization following loss of polarity by apical abscission (*3*), we disrupted ciliary function by chromophore-assisted light inactivation (CALI) (*18*) using the phototoxic fluorescent protein SuperNova (*18*, *19*) linked to Arl13b. The effect of the singlet oxygen species released by SuperNova following green light irradiation has an effective radius of 3 to 4 nm, which is lower than the mean protein-protein interaction distance in cells (*19*). This technique is therefore likely to result in a specific depletion of the primary cilium membrane–associated Arl13b protein, but not other proteins in close proximity. To verify depletion of Arl13b using this approach, we labeled cells expressing Arl13b-SuperNova, which efficiently localizes to the primary cilium, for Arl13b by immunostaining following green light irradiation on a wide-field imaging system. We observed that green light irradiation for 30 min was accompanied by a complete loss of cilium-localized Arl13b-SuperNova fluorescence (Fig. 3A; *n* = 9 cells, 4 embryos; fig. S3A). Furthermore, this was accompanied by a 60% reduction in Arl13b protein measured using Arl13b antibody (Fig. 3A; *n* = 9 cells, 4 embryos; fig. S3B) compared with cells not subjected to green light irradiation (*n* = 11 cells, 2 embryos). We further demonstrated that the centrosome remains unaltered (Fig. 3B; *n* = 9 cells, 4 embryos; movie S10), and EB3-GFP comets continued to extend from this organelle following CALI (Fig. 3, C and D; 18 of 18 cells, 11 embryos; movies S11 and S12), confirming that the microtubule nucleating capacity of the centrosome remained unaltered after this procedure. We then disrupted the Arl13b^+^ particle in proximal cells that were transfected with Arl13b-SuperNova and GFP-GPI. Following green light irradiation, these cells retracted their apical process normally (Fig. 3E; *n* = 14 of 15 cells, 12 embryos; movie S13) but were unable to initiate axonogenesis (*n* = 5 of 6 cells, 5 embryos), suggesting that the remodeled primary cilium directs this process. We then disrupted the remodeled primary cilium in cells undergoing axonogenesis. Following green light irradiation, most cells briefly continued to extend an axon (~3 hours), which subsequently suffered marked collapse (Fig. 3F; *n* = 10 of 14 cells; 11 embryos; movie S14), suggesting that sustained primary cilium–mediated signaling through the remodeled cilium is also required for maintenance of axonogenesis. In contrast, cells transfected only with GFP-GPI, which were subjected to green light irradiation, extended axons normally, demonstrating that this procedure does not generate phototoxicity in the absence of SuperNova (fig. S3C; *n* = 8 of 8 cells, 2 embryos; movie S15). Furthermore, the majority of cells transfected with GFP-GPI and Arl13b-SuperNova, which were not subjected to sustained green light irradiation, extended axons normally (fig. S3D; *n* = 18 of 21 cells, 9 embryos; movie S16), demonstrating that SuperNova does not have phototoxic effects in the absence of sustained green light irradiation. As these experiments were carried out on a wide-field imaging system, there is a possibility that the phenotype observed could be due to depletion of both cilium-localized and undetectable cytoplasmic Arl13b-SuperNova. To discount this possibility, we used a targeted 561-nm laser to disrupt only the ciliary Arl13b of cells expressing Arl13b-SuperNova. Following laser-targeted CALI, we again observed notable axon collapse in an average of 3.5 hours following 561-nm laser irradiation (fig. S4A; *n* = 4 of 5 cells, 5 embryos; movie S17). This was in contrast to cells where the laser was targeted to a cytoplasmic region away from the primary cilium, which continued to extend axons normally (fig. S4B; *n* = 5 of 6 cells, 6 embryos; movie S18). Together, these results strongly indicate that primary cilium–mediated signaling is required for initiation and maintenance of axonogenesis.

To confirm that primary cilium–mediated signaling is directing axon extension following apical abscission, we then disrupted primary cilium function by performing RNA interference (RNAi)–mediated knockdown of Arl13b. Neuroepithelial cells were electroporated with a mix of two short hairpin RNA–expressing constructs targeting different regions of the Arl13b sequence, which also expressed red fluorescent protein (RFP) as a marker for transfected cells (pRFPRNAi Arl13bA and pRFPRNAi Arl13bB). After 26 hours of incubation, embryos were fixed and labeled for Arl13b to assess knockdown efficiency. This revealed a 40% reduction in the number of primary cilia on the electroporated side of the spinal cord versus the unelectroporated side (fig. S5A; *n* = 41 sections of spinal cords, 3 embryos). This was in contrast to control embryos electroporated with a construct targeting firefly luciferase where the number of primary cilia was unaffected (pRFPRNAi luc; fig. S5A; *n* = 9 sections of spinal cords, 3 embryos). We then cotransfected cells with the Arl13b knockdown constructs, GFP-GPI and pCIG-Neurog2, to promote neuronal differentiation. The effect of Arl13b knockdown on axon extension was then monitored by performing time-lapse imaging of these cells. We observed a range of axonogenesis defects in the majority of these cells (*n* = 30 of 41 cells). A subset, which had retracted their apical process, were unable to initiate axonogenesis and remained as round cells with an unpolarized appearance (fig. S5B; *n* = 12 of 41 cells, 10 embryos; movie S19). Another subset of cells, which had initiated axonogenesis, briefly continued to extend their axons, but this was followed by a marked collapse (fig. S5C; *n* = 13 of 41 cells, 10 embryos; movie S20). A further subset of cells had initiated axonogenesis, but these axons partially collapsed, stalled, and did not extend further (*n* = 5 of 41 cells). Only a small proportion of cells were able to extend axons normally for the 10-hour imaging period (*n* = 11 of 41 cells). This was in contrast to cells expressing the control luciferase targeting construct, the majority of which continued to extend their axons throughout the duration of imaging (fig. S5D; *n* = 16 of 19 cells, 7 embryos; movie S21). Together, with the CALI-mediated disruption of ciliary function, these results are consistent with a key role for the remodeled primary cilium in directing initiation and maintenance of axonogenesis in newborn neurons.

### Primary cilium remodeling corresponds with a switch from canonical to noncanonical Shh signaling

**Fig. 4:**
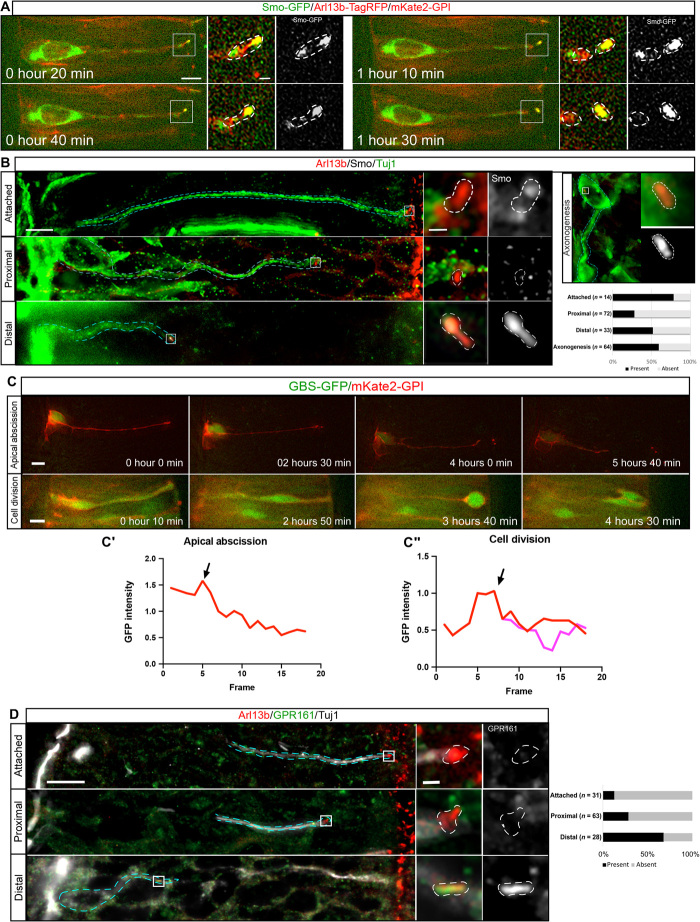
Primary cilium remodeling corresponds with a switch to noncanonical Shh signaling. (**A**) Time-lapse sequence of a cell misexpressing Smo-GFP undergoing apical abscission (movie S22). Insets show an enlarged view of the primary cilium demarcated by white dashed lines. Scale bars, 5 and 0.7 μm (enlarged region). (**B**) Immunostaining to label endogenous Smo (white), Arl13b (red), and Tuj-1 (green). Bottom right corner shows quantification of Smo accumulation in primary cilia at different stages of apical process retraction (bottom right corner). Cyan dashed lines demarcate the differentiating cell, and the inset shows an enlarged view of the primary cilia demarcated by white dashed lines. Scale bars, 5 and 0.7 μm (enlarged region). (**C**) Time-lapse sequence of cells expressing the reporter of Gli activity GBS-GFP undergoing apical abscission (movie S23) and mitosis (movie S24). GFP fluorescence was normalized to the point previous to apical process retraction (C′) and the point of initiation of mitosis (C″). Scale bar, 5 μm. Arrows in C′ and C″ correspond to initiation of apical abscission and initiation of mitosis, respectively. Scale bar, 5 μm. (**D**) Immunostaining to label endogenous GPR161 (green), Arl13b (red), and Tuj1 (white). Right-hand panel shows quantification of GPR161 accumulation in primary cilia at different stages of apical process retraction. Cyan dashed lines demarcate the differentiating cell, and the insets show an enlarged view of the primary cilia demarcated by white dashed lines. Scale bars, 10 and 0.7 μm (enlarged region).

Given the role of the primary cilium in transducing canonical, Gli transcription factor–dependent Shh signals in neural progenitor cells (*16*, *20*, *21*), it is conceivable that the remodeled primary cilium is also transducing Shh signals from surrounding tissues to direct appropriate polarization of differentiating neurons. Shh acts as a chemoattractant for commissural neurons, guiding their axons toward the ventral midline of the spinal cord, through a noncanonical, Gli-independent mechanism (*4*, *5*). However, the mechanisms regulating this switch in the mode of Shh signal transduction are not understood. Furthermore, Arl13b has previously been implicated in controlling entry of the Shh transducer Smoothened (Smo) into the cilium and modulating the chemotactic response of cells to Shh gradients (*22*, *23*). To monitor Shh signaling dynamics during apical abscission, cells were transfected with Smo-GFP and mKate2-GPI and imaged. This revealed that Smo accumulates in the primary cilium in cells poised to undergo apical abscission, and this ciliary Smo was left behind in association with the abscised ciliary particle (Fig. 4A; *n* = 5 cells, 4 embryos; movie S22), consistent with previous experiments (*3*). Furthermore, immunostaining also confirmed that Smo accumulated in the primary cilia of cells that were poised to undergo apical abscission (Fig. 4B; *n* = 11 of 14 cells, 9 embryos). In contrast, Smo did not accumulate in the retained Arl13b^+^ particle of proximal cells (Fig. 4B; *n* = 51 of 72 cells, 9 embryos). Notably, ciliary Smo accumulation was reestablished in distal cells (Fig. 4B; *n* = 17 of 33 cells, 9 embryos), and this was maintained during axonogenesis (*n* = 38 of 64 cells, 9 embryos), indicating reactivation of Shh signaling, which is now transduced by the remodeled primary cilium.

To determine the mode of Shh signaling downstream of Smo during this process, we transfected cells with the reporter of Gli-mediated transcription Gli binding site-GFP (GBS-GFP), which carries eight copies of the Gli-binding site from the FoxA2 enhancer upstream of nuclear GFP (Fig. 4C and movies S23 and S24) (*24*). Imaging these cells revealed that as cells undergo apical abscission, Gli activity was rapidly lost and not reestablished during apical process retraction (*n* = 7 cells, 6 embryos). Conversely, although Gli activity was initially reduced in the progeny of mitotic cells, this was quickly reestablished to premitotic levels (*n* = 10 cells, 6 embryos), confirming that the reduction in Gli activity observed during apical process retraction was not due to GFP photobleaching. The rapid loss of Gli activity during apical abscission indicates an acute cessation of canonical Shh signaling following primary cilium disassembly. Consistent with this, immunostaining for the negative canonical Shh signaling regulator GPR161 (*25*) revealed that GPR161 did not localize to the primary cilium in the majority of Tuj1^+^ cells that were attached to the apical surface (*n* = 27 of 28 cells, 6 embryos; Fig. 4D), indicating active canonical Shh signaling. However, as these cells retracted away from the apical surface, GPR161 localization to the primary cilium progressively increased, with the majority of distal cells now displaying GPR161 accumulation in their primary cilia (*n* = 19 of 28, 6 embryos; Fig. 4D), indicating that canonical Shh signaling is suppressed in these cells.

**Fig. 5:**
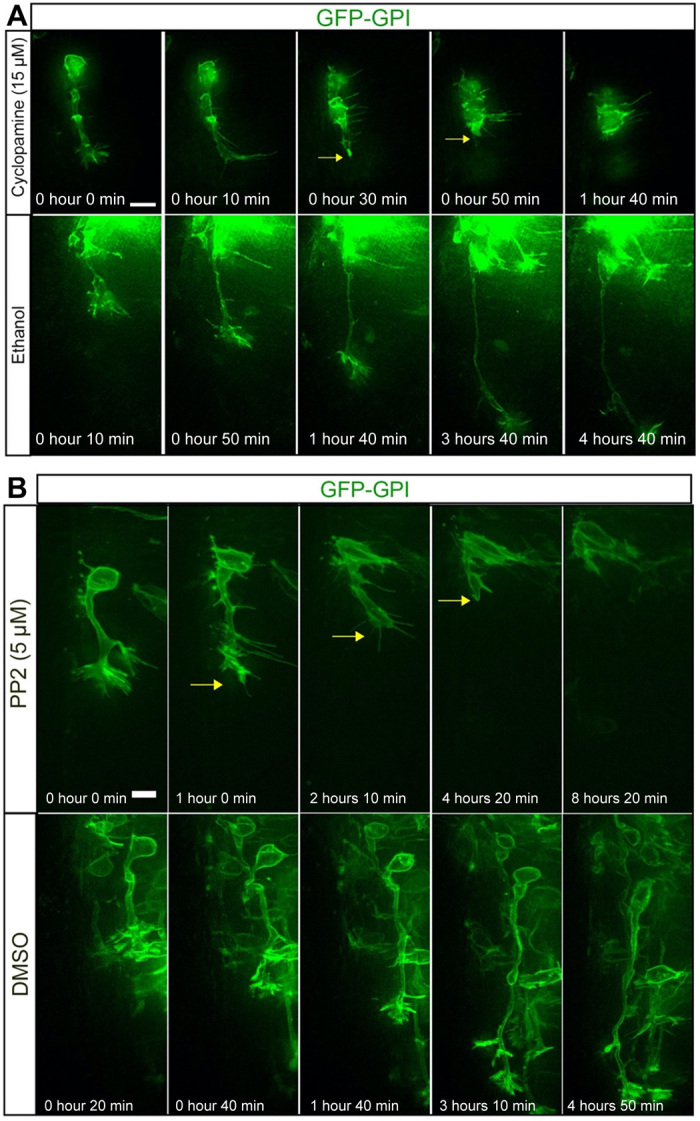
Disruption of noncanonical Shh signaling leads to axon collapse. (**A**) Time-lapse sequence of cell undergoing axonogenesis imaged in medium containing the Smo antagonist cyclopamine (movie S25) and control medium containing ethanol (movie S26). Scale bar, 10 μm. (**B**) Time-lapse sequence of cell undergoing axonogenesis imaged in medium containing the SFK inhibitor PP2 (movie S27) and control medium containing dimethyl sulfoxide (DMSO) (movie S28). Scale bar, 5 μm.

The correlation between cessation of Gli-mediated transcription during apical process retraction and the subsequent initiation of Smo and GPR161 accumulation in the remodeled primary cilium suggests that the remodeled cilium is now transducing Shh through a Gli-independent noncanonical mechanism, which is likely to direct axon extension (*5*). Consistent with this, cells undergoing axonogenesis imaged in medium containing the Smo antagonist cyclopamine (*26*, *27*) abruptly underwent axon collapse within 4 hours of drug application (Fig. 5A; *n* = 17 of 28 cells, 14 embryos; movie S25). This was in contrast to cells imaged in control medium containing vehicle only (ethanol), which continued to extend axons during the 6-hour imaging period (Fig. 5A; *n* = 18 cells, 10 embryos; movie S26). To further confirm the noncanonical mode of Shh signaling in cells extending axons, we then disrupted the function of the Src family kinases (SFKs), which mediate the cytoskeletal rearrangements downstream of noncanonical Shh signaling, using the chemical inhibitor PP2. We observed that cells extending axons imaged in medium containing PP2 also abruptly collapsed their axons (Fig. 5B; *n* = 19 of 20 cells, 6 embryos; movie S27), in contrast to cells imaged in medium containing vehicle control dimethyl sulfoxide (DMSO) (Fig. 5B; *n* = 17 of 22 cells, 6 embryos; movie S28), which continued to extend axons during the 8-hour imaging period. Together, these results indicate that cilium disassembly provides a pause in Gli-dependent Shh signaling as the newborn neuron exits the cell cycle. Subsequent cilium remodeling in the newborn neuron, which has now transitioned from a cycling progenitor to a postmitotic neuron but is encountering the same signaling cues, facilitates entry of GPR161 and Smo to the remodeled cilium, leading to a switch to Smo-dependent Shh transduction in the absence of Gli-mediated transcription to direct axon extension.

## DISCUSSION

**Fig. 6:**
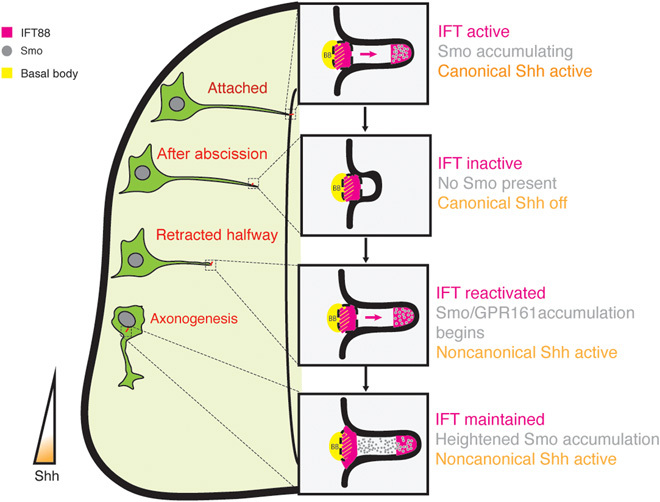
Final model. Remodeling of the primary cilium during neuronal differentiation directs the transition from canonical to noncanonical Shh signaling.

This work uncovers a previously undiscovered mechanism that facilitates a switch in the response to the signaling environment as cells undergo differentiation (Fig. 6). Neural progenitor cells have an apical primary cilium that is required for transduction of canonical, Gli transcription factor–dependent Shh signaling, which maintains these cells as cycling progenitors. As neuronal differentiation proceeds, newborn neurons undergo primary cilium disassembly and begin to delaminate from the neuroepithelium. We show that this step results in the inheritance of a small Arl13b^+^ particle, which contains an axoneme and is accompanied by down-regulation of Gli transcriptional activity, indicating that primary cilium disassembly mediates an acute cessation of canonical Shh signaling. This further raises the possibility that primary cilium disassembly also facilitates an overall resetting of the signaling state of these cells. As the newborn neuron retracts its apical process to delaminate from the neuroepithelium, the retained Arl13b^+^ particle progressively elongates to generate a remodeled primary cilium. This remodeling is characterized by a progressive restoration of IFT from the point of mid-retraction, strongly indicating that cells at this point of differentiation are once again competent to sense extracellular signaling cues. This concept is confirmed by our observation that ciliary Smo accumulation is restored at the mid-retraction point. Notably, this corresponds with ciliary accumulation of the negative canonical Shh regulator GPR161 in the absence of Gli transcriptional activity. Hence, primary cilium remodeling results in a reconfigured signaling center, which now facilitates transduction of Shh signals from the same extracellular source, but in a noncanonical, Gli transcription–independent manner. It is now important to understand how primary cilium remodeling regulates this switch in the mode of Shh signaling. Our finding that the negative Shh regulator GPR161 accumulates in the remodeled primary cilium gives a clue as to how this switch may operate by suggesting that the remodeled primary cilium has a distinct molecular composition that permits specific modes of signal transduction. Future studies on the mechanisms facilitating GPR161 accumulation in the remodeled primary cilium and how this leads to noncanonical Shh signal transduction in cooperation with Smo are now required to understand how this switch operates. Furthermore, future studies on the overall molecular composition of the original versus remodeled primary cilia will determine how differentiating cells integrate and dynamically switch their response to the common signaling environment.

Our observations that compromising the function of the remodeled primary cilium or inhibiting the key components of noncanonical Shh signal transduction (Smo and SFK) leads to loss of axon extension provide an unexpected role for the primary cilium in controlling initiation and maintenance of axon extension through transduction of noncanonical Shh signaling. Although it is widely accepted that axon extension is modulated by noncanonical Shh signaling, the precise molecular mechanisms of this signal transduction pathway have remained elusive. Our work provides a starting point for future studies into the molecular cascades downstream of the remodeled primary cilium and how these directly facilitate the cytoskeletal remodeling events that drive the process of axonogenesis in early differentiating neurons.

On a wider scale, dynamic changes in the molecular configuration of primary cilia may also regulate similar switches in interpretation of other signaling molecules during neuronal differentiation and in other developmental processes such as gastrulation and neural crest delamination. Furthermore, abnormal activation of canonical Shh signaling through the primary cilium is associated with a number of cancer types (*28*). Our findings raise the possibility that errors in primary cilium remodeling events may lead to inappropriate interpretation of Shh signals, resulting in a disruption of the balance between proliferation and differentiation. It is therefore also important to investigate whether similar primary cilium remodeling events are a feature of early tumor formation.

## MATERIALS AND METHODS

### In ovo electroporation and plasmids

Fertilized chicken (*Gallus gallus domesticus*) eggs were obtained from Henry Stewart & Co. Ltd. (Louth, Lincolnshire, LN11 9GN, UK) and incubated at 38°C to Hamburger and Hamilton stage HH10-12. Spinal cords were electroporated as described previously (*9*), with the lowest possible concentration of plasmid DNA that allowed visualization of the marker being analyzed, typically ranging from 25 to 400 ng/μl. The following plasmids were used: pCAG-2A-Arl13b-TagRFP, pCMV-mNeonGreen-IFT88, pCMV-Arl13b-SuperNova, pCMV-PactYFP, pCMV-PactTagRFP, pCAGGS-GFP-GPI, pCAGGS-mKate2GPI, pCMV-mNeonGreen-EB3, pCS+-Arl13b-GFP (a gift from B. Ciruna), pRFPRNAiCLuc, pRFPRNAiCArl13bA, pRFPRNAiCArl13bB, and pEGFP-mSmo (no. 25395, Addgene). pCMV-Arl13b-SuperNova was generated by inserting Arl13b and SuperNova into pCMV plasmid. Arl13b was amplified from pCS+-Arl13b-GFP with primers 5′-GAA GGC TAG CAT GTT CAG TCT GAT GG-3′ (Arl13bF) and 5′-GAA GGT CGA CTG AGC ATC ACT GTT AGG T-3′ (Arl13bR). SuperNova was amplified from pRSETB-SuperNova (no. 53234, Addgene) using primers 5′-GAA GAC CGG TCG CCA CCA TGG GTT CAG AGG TCG-3′ (SuperNovaF) and 5′-GAA GGC GGC CGC TTA ATC CTC GTC GCT A-3′ (SuperNovaR). To generate pCMV-mNeonGreen-IFT88, we used primers 5′-CCC CCC AAG CTT ATG GTG AGC AAG GGC GA-3′ (NeonGreenF) and 5′-CGG CGG GGT ACC CTT GTA CAG CTC GTC CAT G-3′ (NeonGreenR) to amplify mNeonGreen and 5′-CGG CGG GGT ACC ATG GAA AAT GTT CAT CTG GC-3′ (Ift88F) and 5′-AAG GAA AAA AGC GGC CGC CTA TTC TGG GAG CAA GTC A-3′ (Ift88R) to amplify Ift88 from EF5-FRT-TagRFP-T-IFT88 (no. 61684, Addgene).

### Immunofluorescence and fixed tissue imaging

Chick embryos [between embryonic day 3 (E3) to E4] were fixed in 4% formaldehyde and dehydrated in 30% sucrose overnight. These were then mounted in 1.5% Luria-Bertani (LB) agar, dehydrated in 30% sucrose again, and then snap frozen on dry ice. Cryosections of 20-μm thickness were then collected using a Leica CM3050s cryostat and then processed for immunofluorescence. For immunofluorescence, samples were permeabilized with 0.1% Triton X-100 (Sigma-Aldrich) and blocked in 1% donkey serum (Sigma-Aldrich). Primary antibodies were used at the following dilutions: Tuj1 (80120, BioLegend) 1:1000, Arl13b (17711-1-AP, Proteintech), Smo (20787-1-AP, Proteintech) 1:50, IFT88 (13967-1-AP, Proteintech) 1:200, and GPR161(13398-1-AP, Proteintech). All secondary antibodies were Alexa Fluor conjugates (Life Technologies) and used at 1:500. Images were acquired using a 60× 1.40 numerical aperture (NA) objective on a Zeiss Cell Observer Z1 microscope system (Carl Zeiss). 3D images of the primary cilium were acquired using a Zeiss LSM 880 Airyscan system equipped with a 60× 1.40 NA objective.

### Embryo slice culture and time-lapse imaging

Embryos were electroporated at Hamburger and Hamilton stage HH10-12, incubated for 16 hours, sliced, and embedded in type I collagen (Corning) on a glass-bottomed dish (FluoroDish, World Precision Instruments), as described previously (*9*). Slices were taken from the trunk region between the wing and leg buds. Embedded slices were allowed to recover for an hour in neurobasal medium (Thermo Fisher Scientific) supplemented with B27 (Thermo Fisher Scientific) and GlutaMAX (Gibco) before imaging was started. For inhibitor experiments, the medium was replaced with medium containing 15 μM cyclopamine (Stratech Scientific) or 5 μM PP2 (Merck) or carrier control ethanol (Merck) or DMSO (Life Technologies) at the start of imaging. Slices were imaged using a Zeiss Cell Observer Z1 system enclosed in an environment chamber maintained at 37°C and 5% CO_2_. Image acquisition was carried out with a 40× 1.2 NA silicone immersion objective (Carl Zeiss), a Colibri 7 light-emitting diode (LED) light source (Carl Zeiss) and a Flash4 v2 sCMOS camera (Hamamatsu). Image stacks were acquired using minimal exposure times (20 to 50 ms each channel). Stacks consisted of 40 to 50 optical sections (spaced 1.5 μM) using intervals of 10 min between exposures. Imaging was carried out for 4 to 24 hours. To evaluate IFT88 dynamics, differentiating neurons were rapidly imaged at acquisition speed of one z-stack per 10 s for 3 min every hour. For CALI experiments, photobleaching was carried out by irradiating cells with 594-nm illumination from the Colibri 7 LED light source for 15 min. Targeted photobleaching was carried out using a targeted 561-nm laser on a 3i spinning disk confocal enclosed in an environment chamber maintained at 37°C and 5% CO_2_.

### RNA interference

We used a previously described RNAi system (*29*) and GenScript’s small interfering RNA target finder to select target sequences for chicken Arl13b (XM_015297891.2:784-2061). Arl13b target sequences chosen were 5′-GAA GTG GCA AAG GTT GGA GGC T-3′ (pRFPRNAi Arl13b_A) and 5′- GAA GGA AGA AGT GTC CAT GAT G-3′ (pRFPRNAi Arl131b_B). We used Luciferase target sequence 5′-TGC TGC TGG TGC CAA CCC TAT T-3′ as a control as described previously (*29*). Hairpins for the microRNA cloning site were generated by polymerase chain reaction (PCR) using general flanking oligonucleotides (hairpin forward: 5′-GGC GGG GCT AGC TGG AGA AGA TGC CTT CCG GAG AGG TGC TGC CAG TGA GCG-3′; hairpin reverse: 5′-GGG TGG ACG CGT AAG AGG GGA AGA AAG CTT CTA ACC CCG CTA TTC ACC ACC ACC AGT AGG CA-3′) and specific oligonucleotides, which contained the target sequences (Arl13b_A forward: 5′-AGG TGC TGC CAG TGA GCG AAA GTG GCA AAG GTT GGA GGC TTA GTG AAG CCA CAG ATG TA-3′; Arl13b_A reverse: 5′-CAC CAC CAC CAG TAG GCA GAA GTG GCA AAG GTT GGA GGC TTA CAT CTG TGG CTT CAC T; Arl13b_B forward: 5′-AGG TGC TGC CAG TGA GCG AAA GGA AGA AGT GTC CAT GAT GTA GTG AAG CCA CAG ATG TA-3′; Arl13b_B reverse: 5′-CAC CAC CAC CAG TAG GCA GAA GGA AGA AGT GTC CAT GAT GTA CAT CTG TGG CTT CAC T-3′). PCR was performed using 10 ng of each specific oligonucleotide together with 100 ng of the general flanking oligonucleotides. Cycling conditions were set up as described in the KOD Hot Start DNA Polymerase (Merck) manufacturer specifications using 55°C for annealing and 45 s for extension for a total of 30 cycles. PCR products were gel purified, digested with Nhe I and Mlu I, and subcloned into pRFPRNAiC plasmid.

### Measurements, postprocessing, and statistical analysis

Cilium length measurements for Arl13b and PolyGluTub were carried out using the line tool in Zen 2.3 (blue edition, Carl Zeiss). This measurement was performed using single z-sections selecting only cilia that were completely captured in a single plane. GFP intensity was measured by selecting a circular area (3 μm^2^) in the middle of the cell body and measuring the GFP intensity mean value in every frame. Ciliary IFT88 measurements were carried out using the threshold tool in ImageJ 1.52p (National Institutes of Health, USA) and counting the number of particles and area occupied by IFT88 using the analyze particle function. For cilium number quantification in RNAi experiments, the same tool described above was used to count particles between 0.1 and 1 μm^2^. Arl13b-SuperNova intensity was measured by manually drawing cilia contours and quantifying the intensity mean value in the area selected using Zen 2.3 (blue edition, Carl Zeiss). Deconvolution and postprocessing were performed in Zen 2.3 (blue edition, Carl Zeiss). Images acquired at 1 × 1 binning were deconvolved using the constrained iterative algorithm for a maximum of 40 iterations or until a 0.1% quality threshold was achieved. Statistical analyses were performed in GraphPad Prism version 8.3.0 (GraphPad Software, La Jolla, California, USA). EB3 comet displacement was quantified using the manual tracking option in Imaris version 9.2.1 (Bitplane).

## Supplementary Material

abb0601_Movies_S1_to_S28.zip

abb0601_SM.pdf

## SUPPLEMENTARY MATERIALS

Supplementary material for this article is available at http://advances.sciencemag.org/cgi/content/full/6/21/eabb0601/DC1

## Appendix

View/request a protocol for this paper from *Bio-protocol*.
