# Supplementary material for: Primary cilium remodeling mediates a cell signaling switch in differentiating neurons
Source: Sci Adv. 2020 May 20;6(21):eabb0601. doi: 10.1126/sciadv.abb0601 (PMC7252506; doi:10.1126/sciadv.abb0601)
Supplement: abb0601_SM.pdf [file abb0601_SM.pdf]

Science Advances
AAAS

advances.sciencemag.org/cgi/content/full/6/21/eabb0601/DC1

Supplementary Materials for

Primary cilium remodeling mediates a cell signaling switch in differentiating neurons

Gabriela Toro-Tapia and Raman M. Das*

*Corresponding author. Email: raman.das@manchester.ac.uk

Published 20 May 2020, *Sci. Adv.* **6**, eabb0601 (2020)
DOI: 10.1126/sciadv.abb0601

The PDF file includes:

Figs. S1 to S6
Legends for movies S1 to S28

Other Supplementary Material for this manuscript includes the following:

(available at advances.sciencemag.org/cgi/content/full/6/21/eabb0601/DC1)

Movies S1 to S28

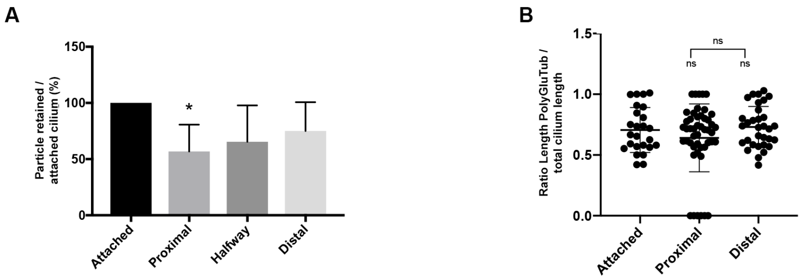

Figure S1: Additional primary cilium length quantifications.
A) Measurement of length of Arl13b^{+} cilia in time-lapse sequences at different stages of apical process retraction relative to the length of the primary cilia of attached cells. This graph corresponds to the data shown in Figure 1B and Movie S2. B) Ratio of the length of the axoneme relative to the primary cilium membrane at different stages of apical process retraction. This graph corresponds to the data shown in Figure 1G.

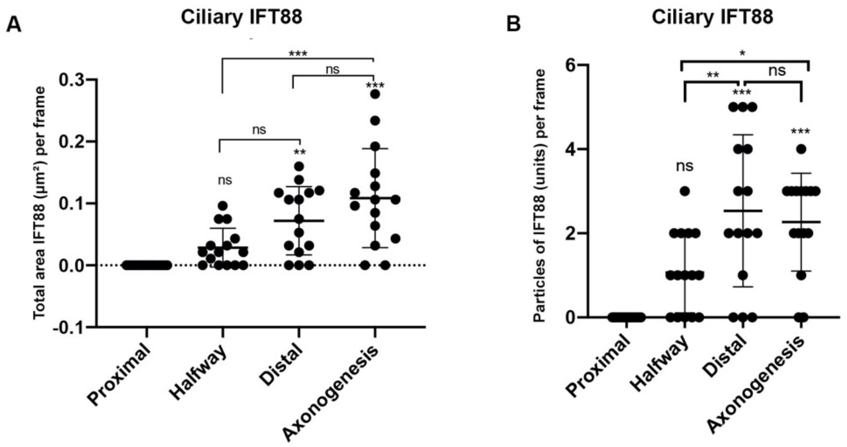

Figure S2: Quantification of IFT88 particles.
A) Quantification of total IFT88 detected in the primary cilium at different stages of apical process retraction and axonogenesis (Mean ± s.d.,ns: not significant, $**P < 0.01$, $***P < 0.001$. Ordinary one-way ANOVA and Turkey's post hoc test used for statistical analyses). B) Number of IFT88 particles present in the primary cilium at different stages of apical process retraction and axonogenesis (Mean ± s.d. ns: not significant, $*<0.05$, $**P < 0.01$, $***P < 0.001$. Ordinary one-way ANOVA and Turkey's post hoc test used for statistical analyses). These graphs correspond to the data shown in Figure 2D and Movies S8-S9.

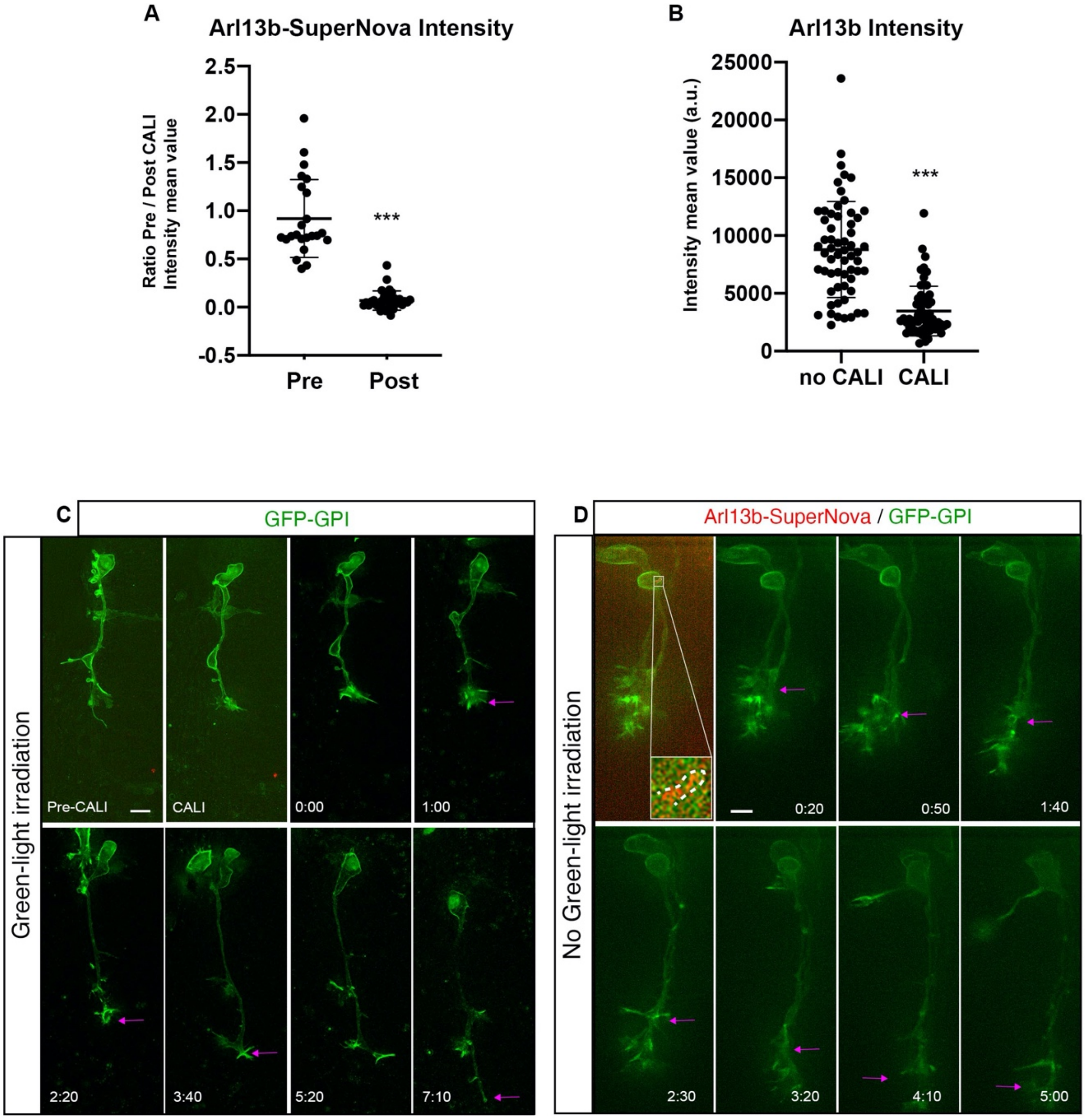

Figure S3: Additional CALI controls.

A) Quantification of Arl13b-SuperNova fluorescence intensity pre and post CALI (Mean ± s.d., ***$P < 0.001$. Unpaired t-test). B) Quantification of fluorescence intensity of endogenous ciliary Arl13b fluorescence in cells either not subjected to green-light irradiation or subjected to sustained green-light irradiation (Mean ± s.d., ***$P < 0.001$. Unpaired t-test). Both graphs refer to data shown in Figure 3A. C) Time-lapse sequence of cell expressing GFP-GPI (green) only to evaluate cell behaviour following green-light irradiation (Movie S15). Scale bar:10µm. D) Time-lapse sequence of cell expressing GFP-GPI (green) and Arl13b-SuperNova (red) not subjected to sustained green-light irradiation (Movie S16). Scale bar: 10µm.

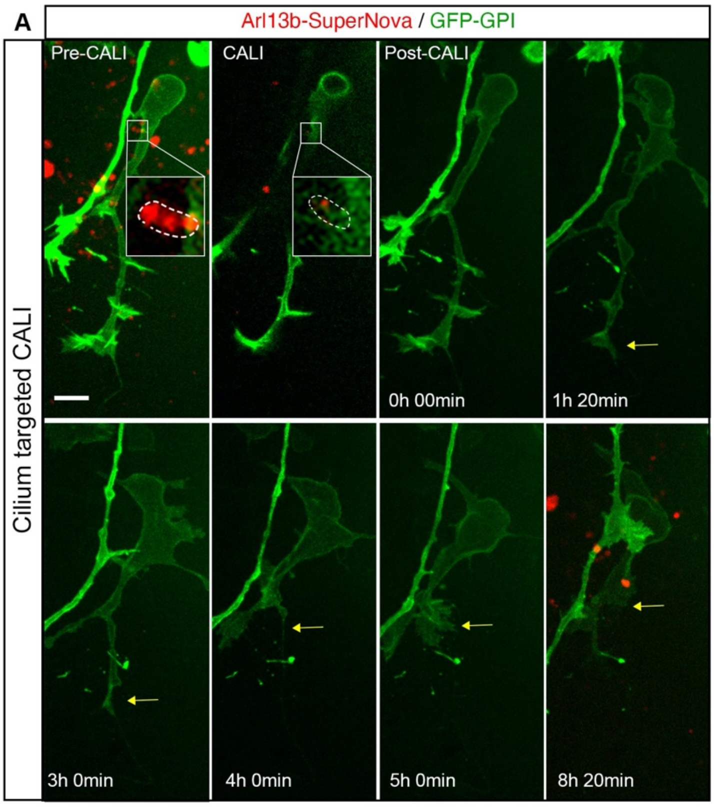

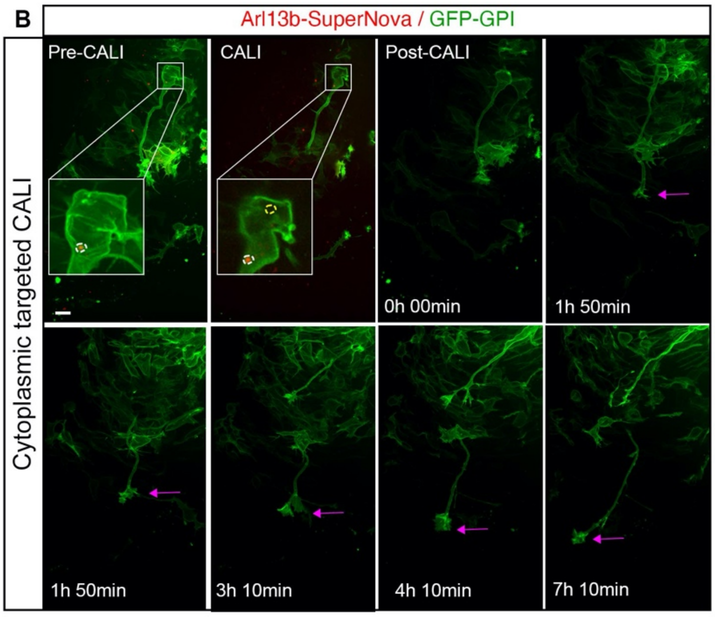

Figure S4: Disruption of the primary cilium by laser-targeted CALI.
A) Time-lapse sequence of cell expressing GFP-GPI (green) and Arl13b-SuperNova (red) (Movie S17). A 561nm laser was used to target only the primary cilium. The inset shows a zoomed-in view of the targeted primary cilium demarcated by white dashed lines. Scale bar: 10µm. B) Time lapse sequence of a cell expressing GFP-GPI (green) and Arl13b-SuperNova (red) (Movie S18). A 561nm laser was used to target a non-ciliary cytoplasmic region of the cell body. The inset shows a zoomed-in view of the cell body with the primary cilium demarcated by white dashed lines and the targeted cytoplasmic region by yellow dashed lines. Scale bar: 10µm.

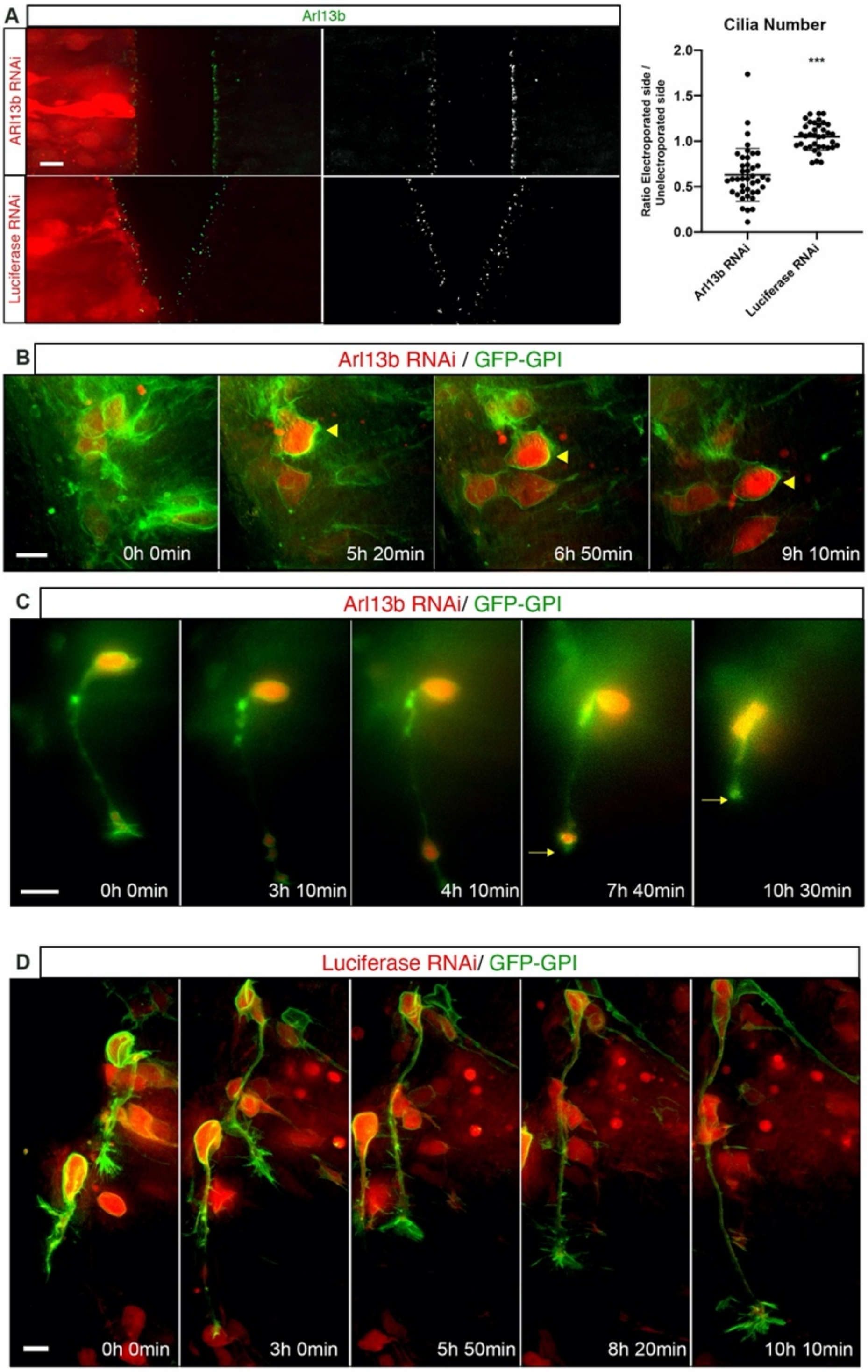

Figure S5: RNAi-mediated knockdown of Arl13b leads to defects in axon extension.
A) Spinal cord tissue electroporated with a mix of two RNAi constructs targeting Arl13b (Arl13b-RNAi) or a construct targeting firefly luciferase (Luciferase RNAi). Knockdown of Arl13b leads to a reduction in the number of Arl13b-labelled primary cilia (green) on the electroporated side compared to the unelectroporated side. Graph shows quantification of the ratio of primary cilia on the electroporated versus unelectroporated side (Mean ± s.d., ****P* < 0.001. Unpaired t-test). B) Time-lapse sequence of cells expressing GFP-GPI (green) and Arl13b-RNAi (red) that have not started to extend axons. Scale bar: 10µm. C) Time-lapse sequence of cells expressing GFP-GPI (green) and Arl13b-RNAi (red) that had started extending axons. Scale bar: 10µm. D) Time-lapse sequence of cell expressing GFP-GPI (green) and Luciferase-RNAi (red) extending axon. Scale bar: 10µm.

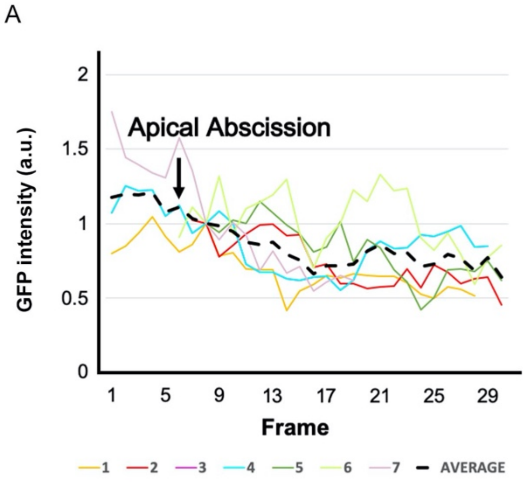

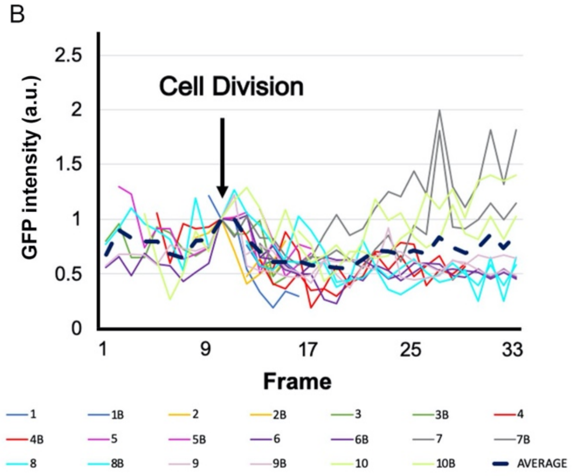

Figure S6: Additional quantification of GBS-GFP fluorescence intensities.
A) Quantification of GFP fluorescence intensity in seven cells undergoing apical abscission. Black dashed line represents median fluorescence intensity. B) Quantification of GFP fluorescence intensity in ten cells undergoing mitosis. Black dashed line represents median fluorescence intensity.

Movie Legends

Movie S1: ***Arl13b⁺ particle retention following apical abscission.***
Time-lapse sequence showing a cell undergoing apical abscission. Arl13-TagRFP labels the ciliary membrane (red) and GFP-GPI the cell membrane (green). Time (hours : minutes).

Movie S2: ***Primary cilium reassembly during neuronal differentiation.***
Time-lapse sequence of a cell undergoing apical process retraction. Arl13-TagRFP labels the ciliary membrane (red) and GFP-GPI the cell membrane (green). Time (hours : minutes).

Movie S3: ***The reassembled primary cilium is maintained in cells undergoing axonogenesis.***
Time-lapse sequence of a cell undergoing axonogenesis. Arl13-GFP labels the ciliary membrane (green) and mKate2-GPI the cell membrane (red). Neurog2-GFP was co-transfected to accelerate neurogenesis in the spinal cord. Time (hours : minutes).

Movie S4: ***IFT88 localises to the base of the Arl13⁺ particle during early stages of apical process retraction.***
Immunostaining to label endogenous IFT88 (green), endogenous Arl13b (red) and Tuj1 (white) followed by Airyscan enhanced-resolution microscopy and 3D reconstruction.

Movie S5: ***IFT88 begins to accumulate in the tip of the reassembled primary cilium in cells halfway through apical process retraction.***
Immunostaining to label endogenous IFT88 (green), endogenous Arl13b (red) and Tuj1 (white) followed by Airyscan enhanced-resolution microscopy and 3D reconstruction.

Movie S6: ***IFT88 accumulation to the tip of the reassembled primary cilium continues in cells distal to the apical surface.***
Immunostaining to label endogenous IFT88 (green), endogenous Arl13b (red) and Tuj1 (white) followed by Airyscan enhanced-resolution microscopy and 3D reconstruction.

Movie S7: ***IFT88 accumulation to the tip of the reassembled primary cilium continues during axonogenesis.***
Immunostaining to label endogenous IFT88 (green), endogenous Arl13b (red) and Tuj1 (white) followed by Airyscan enhanced-resolution microscopy and 3D reconstruction.

Movie S8: ***Intraflagellar trafficking is re-established during apical process retraction.***
Time-lapse sequence of differentiating neuron undergoing apical process retraction. IFT88-mNeonGreen was used to label anterograde intraflagellar transport (green), Arl13b-TagRFP to label ciliary membrane (red) and mKate-GPI to label the cell membrane (red). Arrowhead indicates primary cilium enlarged in the right-hand panel. Time (hours : minutes). Enlarged region displays time in seconds.

Movie S9: ***Maintenance of Intraflagellar trafficking in cell undergoing axonogenesis.***
Time-lapse sequence of differentiating neuron undergoing axonogenesis. IFT88-mNeonGreen was used to label anterograde intraflagellar transport (green), Arl13b-TagRFP to label ciliary membrane (red) and mKate-GPI to label the cell membrane (red). Time (hours : minutes). Enlarged region displays time in seconds.

Movie S10: ***The centrosome remains unaltered following CALI.***
PACT-YFP (green) was used to label the centrosome in CALI experiments and Arl13b-SuperNova to disrupt the primary cilium.

Movie S11: ***EB3-GFP comets continue to extend from the centrosome following CALI in cells attached to the apical surface.***
EB3-mNeonGreen (green) was used to label microtubule plus-ends and Arl13b-SuperNova (red) to target and disrupt the primary cilium.

Movie S12: ***EB3-GFP comets continue to extend from the centrosome following CALI in cells during axonogenesis.***
EB3-mNeonGreen (green) was used to label microtubule plus-ends and Arl13b-SuperNova (red) to target and disrupt the primary cilium.

Movie S13: ***Primary cilium remodelling is required for initiation of axonogenesis.***
Time-lapse sequence of a cell undergoing apical process retraction following CALI-mediated disruption of the retained $Arl13^+$ particle and subsequent cell behaviour. Arl13b-Supernova (red) was used to target and disrupt the primary cilium and GFP-GPI (red) to label the cell membrane. Time (hours : minutes).

Movie S14: ***The remodelled primary cilium is required for maintenance of axonogenesis.***
Time-lapse sequence of CALI-mediated disruption of primary cilium in cell undergoing axonogenesis and subsequent cell behaviour. Arl13b-Supernova (red) was used to target and disrupt the primary cilium and GFP-GPI (red) to label the cell membrane. Time (hours : minutes).

Movie S15: ***GFP-GPI expressing cells extend axons normally following green light irradiation.***
Time-lapse sequence of cells transfected only with GFP-GPI and subjected to green light irradiation during axonogenesis. Time (hours : minutes).

Movie S16: ***GFP-GPI and Arl13b-SuperNova expressing cells not subjected to sustained green light irradiation extend axons normally.***
Time-lapse sequence of cells transfected with GFP-GPI and Arl13b-SuperNova that were not subjected to green light irradiation during axonogenesis. Time (hours : minutes).

Movie S17: ***Primary cilium-targeted CALI leads to axon collapse.***
Time-lapse sequence of a cell misexpressing GFP-GPI and Arl13b-SuperNova subjected to cilium-targeted CALI during axonogenesis. Time (hours : minutes).

Movie S18: ***Cytoplasm-targeted CALI does not alter cell behaviour.***
Time-lapse sequence of a cell misexpressing GFP-GPI and Arl13b-SuperNova and subjected to cytoplasm-targeted CALI during axonogenesis. Time (hours : minutes).

Movie S19: ***Arl13b knockdown disrupts initiation of axonogenesis.***
Time-lapse sequence of cells transfected with GFP-GPI, pRFPRNAi Arl13bA and pRFPRNAi Arl13bB. Time (hours : minutes).

Movie S20: ***Arl13b knockdown disrupts axon extension.***
Time-lapse sequence of cells transfected with GFP-GPI, pRFPRNAi Arl13bA and pRFPRNAi Arl13bB undergoing axonogenesis. Time (hours : minutes).

Movie S21: ***Expression of a luciferase-targeting shRNA construct does not affect axon extension.***
Time-lapse sequence of cells transfected with GFP-GPI and pRFPRNAi-Luciferase undergoing axonogenesis. Time (hours : minutes).

Movie S22: ***Ciliary Smo is left behind in association with the abscised ciliary particle.***
Time-lapse sequence of a cell mis-expressing Smo-GFP and mKate2-GPI undergoing apical abscission. Time (hours : minutes).

Movie S23: ***Cessation of Gli activity following apical abscission.***
Time-lapse sequence of cells expressing the reporter of Gli activity GBS-GFP (green) undergoing apical abscission. mKate2-GPI (red) was used to label the cell membrane. Time (hours : minutes).

Movie S24: ***Gli activity is maintained in cells undergoing mitosis.***
Time-lapse sequence of cells expressing the reporter of Gli activity GBS-GFP (green) undergoing mitosis. mKate2-GPI (red) was used to label the cell membrane. Time (hours : minutes).

Movie S25: ***Axon collapse following inhibition of Smo function by cyclopamine.***
Time-lapse sequence of a cell undergoing axonogenesis imaged in medium containing cyclopamine. GFP-GPI (green) was used to label the cell membrane. Time (hours : minutes).

Movie S26: ***Normal axon extension in medium containing ethanol.***
Time-lapse sequence of a cell undergoing axonogenesis imaged in medium containing ethanol. GFP-GPI (green) was used to label the cell membrane. Time (hours : minutes).

Movie S27: ***Axon collapse following inhibition of SFK function by PP2.***
Time-lapse sequence of a cell undergoing axonogenesis imaged in medium containing PP2. GFP-GPI (green) was used to label the cell membrane. Time (hours : minutes).

Movie S28: ***Normal axon extension in medium containing DMSO.***
Time-lapse sequence of a cell undergoing axonogenesis imaged in medium containing DMSO. GFP-GPI (green) was used to label the cell membrane. Time (hours : minutes)
